# Multi-Scale Microstructural Tailoring and Associated Properties of Press-Hardened Steels: A Review

**DOI:** 10.3390/ma16103799

**Published:** 2023-05-17

**Authors:** Zhuo Cheng, Mengjie Gao, Jinyue Liu, Shuize Wang, Guilin Wu, Junheng Gao, Honghui Wu, Xinping Mao

**Affiliations:** 1 Research Institute for Carbon Neutrality, University of Science and Technology Beijing, Beijing 100083, China; b20200556@xs.ustb.edu.cn (Z.C.); d202110606@xs.ustb.edu.cn (M.G.); s20200330@xs.ustb.edu.cn (J.L.); guilinwu@ustb.edu.cn (G.W.); junhenggao@ustb.edu.cn (J.G.); maoxinping@126.com (X.M.); 2 Beijing Advanced Innovation Center for Materials Genome Engineering, University of Science and Technology Beijing, Beijing 100083, China; 3 Institute for Steel Sustainable Technology, Liaoning Academy of Materials, Shenyang 110004, China

**Keywords:** press-hardened steels, microstructural tailoring, mechanical properties, hydrogen embrittlement, service performance

## Abstract

High-strength press-hardened steels (PHS) are highly desired in the automotive industry to meet the requirement of carbon neutrality. This review aims to provide a systematic study of the relationship between multi-scale microstructural tailoring and the mechanical behavior and other service performance of PHS. It begins with a brief introduction to the background of PHS, followed by an in-depth description of the strategies used to enhance their properties. These strategies are categorized into traditional Mn-B steels and novel PHS. For traditional Mn-B steels, extensive research has verified that the addition of microalloying elements can refine the microstructure of PHS, resulting in improved mechanical properties, hydrogen embrittlement resistance, and other service performance. In the case of novel PHS, recent progress has principally demonstrated that the novel composition of steels coupling with innovative thermomechanical processing can obtain multi-phase structure and superior mechanical properties compared with traditional Mn-B steels, and their effect on oxidation resistance is highlighted. Finally, the review offers an outlook on the future development of PHS from the perspective of academic research and industrial applications.

## 1. Introduction

In modern society, the use of automobiles has grown substantially worldwide, leading to the automotive industry becoming one of the foremost fields of greenhouse gas emissions [1]. This has prompted the industry to confront the significant challenge of achieving carbon neutrality by conserving energy and reducing emissions. Automotive lightweight is a viable strategy for overcoming the dilemma, as every 10% reduction in vehicle weight can lead to a 6–8% fuel consumption reduction and a 5–6% emission reduction [2]. To achieve carbon neutrality, two critical strategies of automotive lightweight are optimizing the vehicle body or using more lightweight materials while ensuring safety, for example, replacing the thick low-strength steels with thin advanced high-strength steels (AHSS) or other lightweight alloys (e.g., Al alloy). Through a comprehensive evaluation of multi-dimensional parameters of structural metallic materials, such as resource reserves, production, unit cost, unit energy consumption, unit greenhouse gas emissions, scrap generation and recycle rate, processing technology, and mechanical performance, etc., steels play a leading role among structural metallic materials and have greater advantages in energy saving and emission reduction throughout the life cycle [3,4,5]. Furthermore, AHSS has the most potential for automotive lightweight in the contemporary era, with its proportion in body-in-white (BIW) determining the level of automobile lightweight [1]. Therefore, the utilization of high-performance AHSS enables sustainable and ecological development in the automobile industry. Nowadays, AHSS is widely used in the automobile industry.

**Figure 1:**
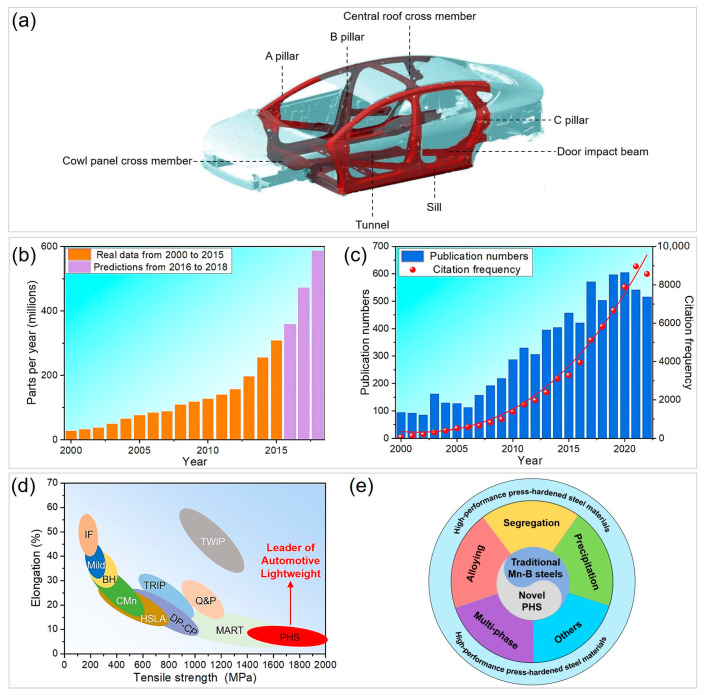
The status of press-hardened steels in industrial application and academic research. (**a**) Applications of hot-stamped parts on automotive body-in-white, (**b**) worldwide demand for hot-stamped parts in the automotive industry, (**c**) publication numbers and citation frequency with the keywords “press hardened steel” or “hot stamping steel” retrieved from Web of Science, (**d**) a comparison of tensile strength and elongation of advanced high-strength steels, indicates press-hardened steels are “the leader of automotive lightweight”, (**e**) application of precipitation, segregation, etc., in traditional Mn-B steels, and the use of Si- or/and Cr alloying, multi-phase design, etc., in novel press-hardened steel, can help tailor the microstructure to obtain improved properties. (**b**) Reprinted with permission from [6].

Press-hardened steels (PHS) have emerged as a crucial material in the field of advanced high-strength steels, receiving increasing attention from both the industrial and academic sectors. PHS are essentially carbon (C-) and manganese (Mn-) alloyed steels with minor Boron (B) addition, guaranteeing high hardenability [6,7,8]. For example, a typical example of PHS is 22MnB5 steel, with a basic chemical composition of 0.19–0.25 wt% C, 1.1–1.4 wt% Mn, and 0.001–0.005 wt% B [6]. Despite the simple composition, the strength of 22MnB5 steel can reach as high as 1400–1700 MPa, with competent ductility of 4–8% [9]. Currently, the competitive cost and high strength advantages have enabled the growing application of PHS in the BIW of automobiles. As shown in Figure 1a, PHS is extensively used as safety-critical components such as A/B/C pillars, door impact beams, sill, tunnel, cross member of the central roof and cowl panel, etc., to “warp” the passengers and improve the overall vehicle crashworthiness in response to frontal and side collision. Dynamic tensile tests also indicate that PHS displays a superior anti-intrusive crash performance to other automotive steels both at low and high strain rates [10]. Although hot-stamped parts have been utilized in the automotive industry since the mid-1980s, rapid development has been witnessed in the past two decades [6]. For instance, the global demand for hot-stamped parts per year has increased from 27.6 million in 2000 to 127.3 million in 2010, and further to a predicted value of 586.2 million in 2018 (Figure 1b) [6]. Meanwhile, the application of PHS in BIW also shows a substantial increase over time, with a maximum proportion of 60% in 2016 [9]. PHS manufacturers include major steel companies worldwide such as Svenskt Stål AB, ArcelorMittal, Baosteel, Schuler, Benteler, and ThyssenKrupp [6]. This bloom in industrial applications has also inspired extensive academic research, as evidenced by the surge in publications and citation frequency related to the keywords “press hardened steel” or “hot stamping steel” in the Web of Science, as shown in Figure 1c. In the past two decades, approximately 7000 related publications with over 65 thousand citations have covered a wide range of topics, including materials science, mechanics, and engineering, among others, thereby promoting the industrialization progress of PHS.

What makes PHS amazing? In addition to the simple chemical composition and low cost, the appreciable strength makes PHS “the leader of automotive lightweight” in the classical strength versus elongation diagram (Figure 1d). Specifically, if one ranks first-generation AHSS grades with tensile strength, the approximate orders from low to high are interstitial-free (IF) steels, mild steels, bake-hardening (BH) steels, carbon-manganese (C-Mn) steels, high-strength low-alloy (HSLA) steels, transformation-induced-plasticity (TRIP) steels, dual-phase and complex-phase (DP-CP) steels, martensitic (MART) steels, and PHS. It is worth noting that PHS also surpasses the strength of second- and third-generation AHSS grades, which include twinning-induced plasticity (TWIP) steels and quench and partitioning (Q&P) steels, respectively [11]. Specifically, PHS can achieve strength from 1400 MPa to above 2000 MPa, which has a broad spectrum of applications in BIW components in the automotive industry.

This review mainly aims to provide insights into the microstructure–property relationship of high-performance PHS materials, which are grouped into traditional Mn-B steels and novel PHS in terms of chemical composition, processing, and microstructural characteristics (Figure 1e). Traditional Mn-B steels have been extensively studied and utilized, as described in [6]. In view of chemical composition, traditional Mn-B steels contain alloying elements such as Si and Cr, but their levels are relatively low, typically less than 0.5% [6,12] (see Table 1), while the novel PHS is featured by increased levels of alloying elements such as Si or/and Cr, as reported in Refs. [13,14]. From the perspective of microstructure, traditional Mn-B steels are usually composed of a single martensitic structure, while novel PHS tends to exhibit a multi-phase structure via tailoring the hot stamping processing route. For traditional Mn-B steels, this review mainly focuses on how microalloying elements help design high-performance PHS, mainly in terms of segregation and precipitation. These properties include mechanical properties, hydrogen embrittlement (HE) resistance, bending properties, impact performance, and others. For novel PHS, this review critically examines the effect of composition and processing optimization on the multi-phase design, mechanical properties, and other service performance, such as oxidation resistance.

**Figure 2:**
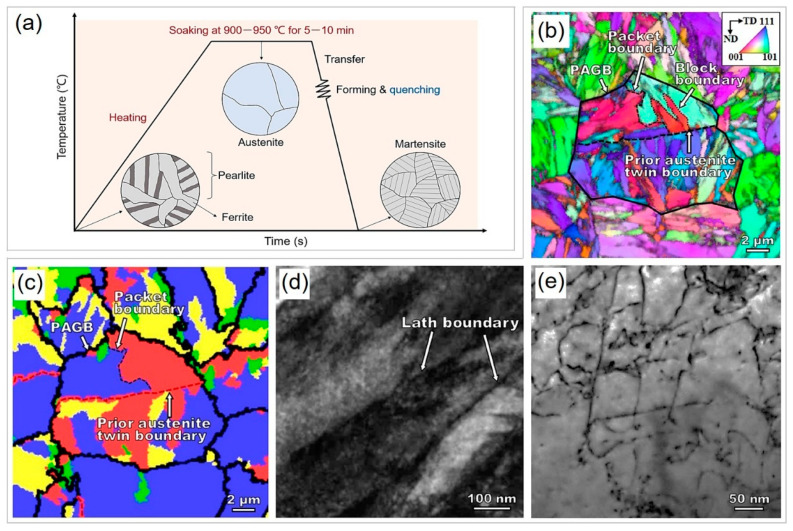
The microstructural evolution and structural features of press-hardened steels. (**a**) Schematic illustration of microstructural evolution during the hot stamping process. (**b**) Typical martensite features of 22MnB5 steel with hieratical structures, including (**c**,**d**) electron backscattering diffraction image showing prior austenite grain boundary, packet boundary, block boundary, (**d**) transmission electron microscopy (TEM) image showing lath boundary, and (**e**) a high density of dislocations inside the lath. Note that (**d**) is the corresponding prior austenite grain and packet reconstruction map of (**c**). (**b**–**e**) Reprinted with permission from [31].

High-strength steels pose significant challenges in conventional cold forming due to their susceptibility to large springback, cracking, and low shape accuracy. However, these challenges can be overcome by hot stamping, which is also referred to as press hardening. This complex forming technology involves elevated temperatures to achieve fine and sophisticated geometries of parts with higher shape accuracy and less springback than cold forming [15]. The hot stamping process is accompanied by a phase transformation. As shown in Figure 2a, the microstructure of as-received PHS is usually comprised of pearlite and ferrite, as it may undergo hot-temperature hot rolling, coiling, cold rolling, and annealing [16]. Before hot stamping, the steel sheet is heated to an austenitizing temperature of 900–950 °C for 5–10 min to fully transform to austenite under the protection of an N_2_ atmosphere; austenite has excellent ductility (>40%) and formability [9,17]. Then, the steel sheet is transferred by the robot arm to a die with cooling systems, where it undergoes hot deformation and quenching with an austenite-to-martensite transition [18]. Generally, bare PHS steel plates require Al-Si or Zn coatings to protect against surface oxidation during the transfer process from the heating furnace to the die [9,19]. For most research on PHS, the direct austenitizing and quenching processes are used to simulate the hot stamping process because carrying out real hot stamping is challenging in laboratory conditions [20]. After the hot-stamped parts are assembled to BIW, a paint baking process is usually conducted at 160–190 °C for 15–60 min [21]. This process is similar to low-temperature tempering, which increases the yield strength and reduces the tensile strength to varying degrees, but does not greatly affect the martensitic structure of PHS [22]. For high-strength PHS, low-temperature tempering can improve ductility due to the annihilation of high-density dislocations, while the precipitation of nanoparticles can maintain the high strength [23]. It should be noted that the actual hot stamping process is complex, as it couples phase transformation and plastic deformation [24]. To better predict the multi-phase transformation process during hot stamping, various simulations including two-dimensional representative volume element models [25], computer-aided design mode [26,27], numerical simulation [28], and finite element method [29] are conducted in addition to experimental verification.

Similar to typical carbon steels, the martensitic microstructure of PHS exhibits a hierarchical structure [30]. For instance, classical 22MnB steel displays a multi-scale microstructure that includes prior austenite grain boundary (PAGB), packet boundary, block boundary, and lath boundary, as illustrated in Figure 2b–e [31]. A fine block boundary plays a vital role in limiting dislocation motion and refining grain boundaries [32]. It is reported that the grain refinement effect of PHS can achieve 100–200 MPa [33,34]. In addition to multiple interfaces, a high density of dislocations at the level of 10^15^ m^−2^ is another significant feature, and this provides a maximum strengthening effect for PHS. Specifically, dislocation strengthening can contribute to a strength increase of 700–1000 MPa [20,34]. Hence, as with most martensitic steel, high-density dislocations are crucial to the attainment of high strength [35]. In addition to grain refinement strengthening and dislocation hardening, the main strengthening mechanisms are precipitation hardening, principally from microalloying carbides and solid-solution strengthening predominantly caused by interstitial C element [20]. Therefore, the high strength of PHS results from the synergistic effect of multiple strengthening mechanisms.

## 2. Traditional Mn-B Steels

### 2.1. Development Trend

At present, 1500 MPa grade 22MnB5 steel is widely used in BIW applications. Nevertheless, the need for enhanced strength and ductility has propelled the development of high-performance PHS [36,37]. This can satisfy the increasing demand for lightweight materials in the automotive industry, especially for emerging new-energy vehicles, as their power system is typically less powerful than that of the gasoline car. Over the past few decades, PHS with higher strength levels have been continuously developed. For martensitic steels, the interstitial element C plays a critical role in improving strength [32,38]. It is reported that increasing the C content in martensite not only refines the structure but also enhances the dislocation density [32,35], resulting in both grain refinement strengthening and dislocation hardening, thus increasing the overall strength level. Generally, the steel grade and strength level of traditional Mn-B steel is determined by the C content, as presented in Table 1. For example, 30MnB5 and 34MnB5 have C contents of 0.30–0.31 wt% and 0.33–0.34 wt%, respectively, and their corresponding tensile strength can reach 1880 MPa and 1980 MPa [20,39]. More recently, there have also been considerable research efforts devoted to developing PHS with even higher strength, such as 0.38% C content corresponding to a tensile strength of 2181 MPa [10].

**Table 1:** Chemical compositions and tensile strength of traditional Mn-B steels.

Steel	Chemical Composition (wt%)									Tensile Strength (MPa)	Ref.
	C	Mn	B	Si	Cr	Ti	Nb	V	Mo		
22MnB5	0.23	1.23	0.004	0.21	0.29	0.037	/			1463	[12]
25MnB5	0.25	1.20	0.003	0.18	0.25	0.033	/			1491	[12]
29MnB5	0.29	1.26	0.004	0.18	0.20	0.03	/			1741	[12]
30MnB5	0.3	1.58	0.005	0.33	0.36	0.055	/	0.04		1880	[20]
32MnB5	0.32	1.2	0.003	0.25	0.12	0.03	0.05			1904	[40]
34MnB5	0.34	1.38	0.002	0.29	0.02	0.02	/	/	0.29	1980	[39]
35MnB5	0.36	1.4	0.003	0.2	/	/	/	/	/	2000	[41]
38MnB5	0.38	1.2	0.003	0.19	0.28	0.024	/	/	0.005	2181	[10]

PHS exhibits a remarkable increase in strength as the carbon content increases. However, the trade-off for such enhanced strength is a decrease in ductility, as depicted in Figure 1d. This trend is more evident at higher levels of strength [42]. Such high-strength PHS with limited ductility fails to meet the requirements for crash safety and energy absorption in the current BIW design. Furthermore, HE and weldability may be more severe at enhanced strength and C levels. Recent research has uncovered a simple yet highly effective approach to optimizing the mechanical properties, HE resistance, and weldability of PHS. This method, called microalloying technology [43,44], alters the microstructure of PHS, resulting in significant improvements in the aforementioned properties.

### 2.2. Effect of Microalloying Elements on Mechanical Performance Improvement in PHS

**Figure 3:**
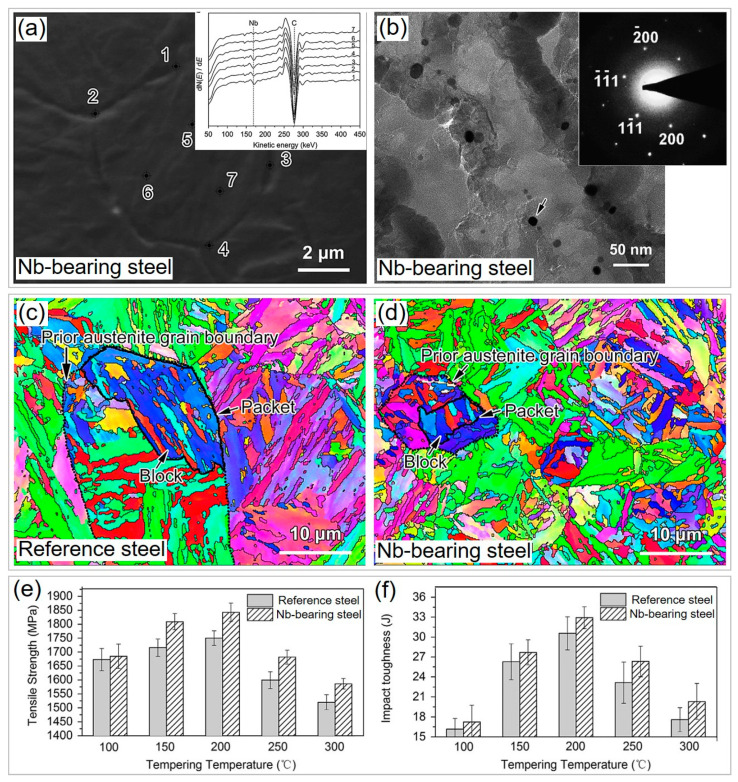
Structural refinement and improved properties of 1800 MPa grade 30MnB5 steel via microalloying technology. (**a**) Scanning electron microscopy (SEM) secondary electron image of prior austenite grain boundary of Nb-bearing steel showing segregation of Nb as revealed by Auger electron spectroscopy (inset). (**b**) Formation of massive Nb-bearing nanoprecipitates in Nb-bearing steel revealed by TEM image with an electron diffraction pattern of Nb-containing particle pointed by black arrow (inset). EBSD images of (**c**) reference steel and (**d**) Nb-bearing steel indicate Nb microalloying can refine martensite structure. A comparison of (**e**) tensile strength and (**f**) impact toughness of reference and Nb-bearing steels at various tempering temperatures. Reprinted with permission from [48].

Microalloying elements, such as Ti, Nb, V, and Mo, are commonly added to produce PHS, which causes a limited effect on production cost for trace amounts of addition. These elements are strong carbide formers [45,46] and tend to segregate [47] at interphase, which can cause a precipitation-strengthening effect and solute drag in PHS [48]. In our recent work, the precipitation behavior of microalloying elements in PHS is unveiled [20]. It is found that the microalloyed carbides mainly precipitate during hot rolling and coiling, as a result of the low solid solubility of microalloying carbides in ferrite. Although many carbides dissolve during austenitizing, a large number of carbides remain in the martensite after quenching. The addition of microalloying elements can improve the microstructure and properties of PHS. For example, Wu et. al. [48] investigated the effect of 0.021% Nb microalloying on the microstructure and mechanical properties of baseline 30MnB5 steel. It was found that Nb segregates at the prior austenite grain boundary and forms a large amount of Nb-bearing precipitates (Figure 3a,b). As a result, the martensite structure of Nb-bearing steel is significantly refined relative to reference steel (Figure 3c,d). This is because the grain growth of austenite is confined by the drag of Nb solute atoms and the pinning of Nb-bearing precipitates. Similarly, Li et al. [49] also reported that the addition of Nb can simultaneously refine prior austenite grain, packet, and lath. As a consequence, the Nb-bearing steel exhibits higher tensile strength and better impact toughness than reference steel after exposure to low-temperature tempering (Figure 3e,f), which is attributed to the refined martensite structure and Nb-containing precipitates. Microalloying carbides can impede dislocation motion and enhance the work-hardening ability of martensite, causing a considerable strengthening effect of 100–300 MPa [34].

In most cases, various kinds of microalloying elements are co-added in PHS, instead of one element. The effect of such microalloying elements on the microstructure and mechanical properties of PHS is summarized in Table 2. Regardless of the steel type, the increased addition of microalloying elements can contribute, to varying degrees, to the structural refinement of martensite considering prior austenite grain size. In some cases, this can lead to an improvement in the strength–ductility balance, such as with Nb- and Mo-microalloyed 22MnB5 steel [49], Ti- and Nb-microalloyed 22MnB5 steel [50], Ti-, Nb- and V-microalloyed 22MnB5 steel [51], Ta-microalloyed 22MnB5 steel [52], and Nb- and Mo-microalloyed 38MnB5 steel [53]. However, simply increasing the content of microalloying elements cannot necessarily achieve strength–ductility synergy. For 0.03% Ti-bearing 22MnB5 steel, the tensile strength increases by 38.2 MPa at 0.022% Nb addition, but decreases by 88.5 MPa at 0.078% Nb addition [54]. Furthermore, other examples also show that increasing microalloying elements may harm mechanical properties [34,40,55,56]. Recent work on V-bearing PHS has shown that increasing the V content from 0 to 0.2% can consume more C in the solid solution of martensite matrix and cause precipitate coarsening, resulting in reduced strength [20]. Additionally, Zhang et al. [54] suggested that the grain refinement resulting from microalloying carbonitride is another critical factor affecting strength.

### 2.3. Effect of Microalloying Elements on Hydrogen Embrittlement of PHS

During production and service, H atoms tend to diffuse into steel and accumulate at defects and interfaces with stress concentration, such as micro-voids, grain, and phase boundaries, resulting in reduced ductility or/and strength [57]. It is commonly accepted that HE sensitivity increases with strength [58,59,60]. PHS, as one of the typical high-strength martensitic steels, is particularly sensitive to HE, which poses a significant challenge to its widespread application [61,62]. Consequently, the development of PHS with higher strength has to tackle the dilemma of HE to meet safety requirements.

**Figure 4:**
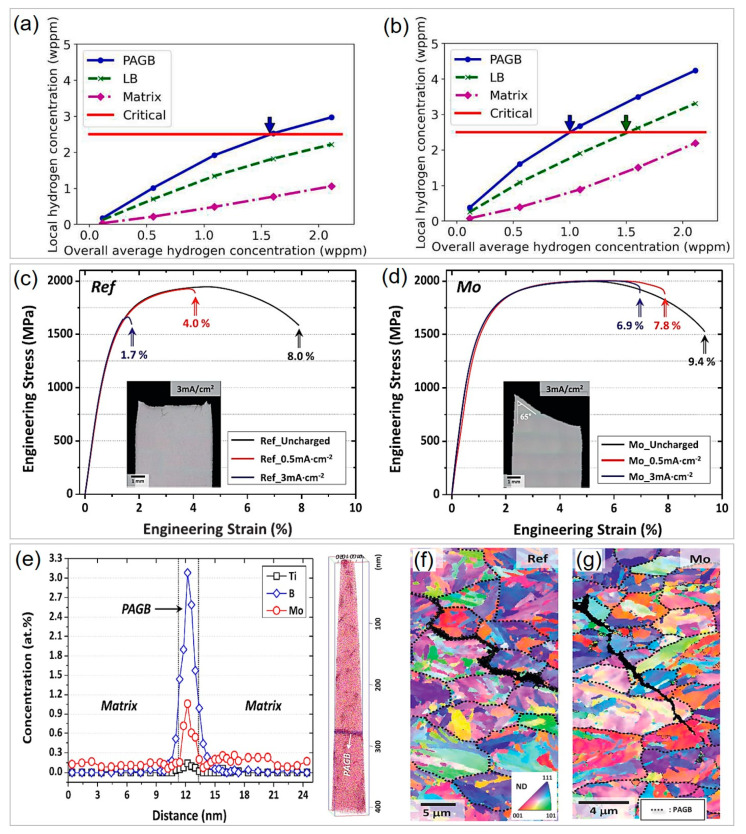
Mechanisms for hydrogen embrittlement of press-hardened steels and enhancement of hydrogen embrittlement resistance of 32MnB5 steel via microalloying. Relationship between local hydrogen concentration in prior austenite grain boundary (PAGB) and lath boundary (LB), as well as the martensite matrix and the overall average hydrogen concentration at (**a**) low hydrostatic stress region and (**b**) high hydrostatic stress region. The red line in (**a**,**b**) refers to the local critical hydrogen concentration above which cracking can occur as indicated by the colored arrows. Reprinted with permission from [63]. (**c**,**d**) Engineering stress–strain curves of the slow-strain-rate tensile test before and after H charging at current densities of 0.5 and 3 mA·cm^−2^ for the (**c**) reference and (**d**) Mo-bearing steels with fracture images (insets). (**e**) APT characterization indicating Mo segregation at prior austenite grain boundary for Mo-bearing steel. EBSD of deformed samples showing the fracture mode of (**f**) transgranular fracture for reference steel and (**g**) intergranular fracture for Mo-bearing steel after slow-strain-rate tensile testing at the current density of 3 mA·cm^−2^. Reprinted with permission from [56].

In martensitic steels, PAGB is pivotal for intergranular failure after being subjected to hydrogen charging. Combining the phase field model for martensitic transformation with a modified hydrogen diffusion model, Ngiam et al. [63] found that PAGB is the primary site in trapping hydrogen. As shown in Figure 4a,b, high hydrostatic stress can trigger hydrogen aggregation, and the local hydrogen concentration on PAGB is consistently higher than the lath boundary and martensite matrix at a fixed overall average hydrogen concentration. When the local hydrogen concentration at PAGB exceeds the critical value, intergranular cracking along PAGB occurs due to reduced cohesive strength, which follows the classical hydrogen-enhanced decohesion theory [64,65]. In contrast to hydrogen, the segregation of other elements such as Mo can help enhance the grain boundary cohesion and thus HE resistance [66,67]. Yoo et al. [56] investigated the effect of Mo on the HE resistance of 1900 MPa grade 32MnB5 steel. The elongation loss of the slow-strain-rate tensile tests before and after H charging is generally used to evaluate the HE resistance index, and can be calculated by (δ_uncharged_ − δ_charged_)/δ_uncharged_, where δ_uncharged_ and δ_charged_ are the tensile elongations of the H-uncharged and H-charged specimens, respectively. As demonstrated in Figure 4c,d, slow-strain-rate tensile tests revealed that Mo-bearing steel shows much lower elongation loss (17–26%) than the reference steel without Mo (50–79%). Atom probe tomography (APT) characterization detected Mo atoms segregation at the PAGB for Mo-bearing steel (Figure 4e), and the fracture mode changed from transgranular fracture for reference steel to intergranular fracture for Mo-bearing steel (Figure 4f,g), indicating that the enhanced PAGB cohesion due to Mo segregation can significantly reduce HE susceptibility.

**Figure 5:**
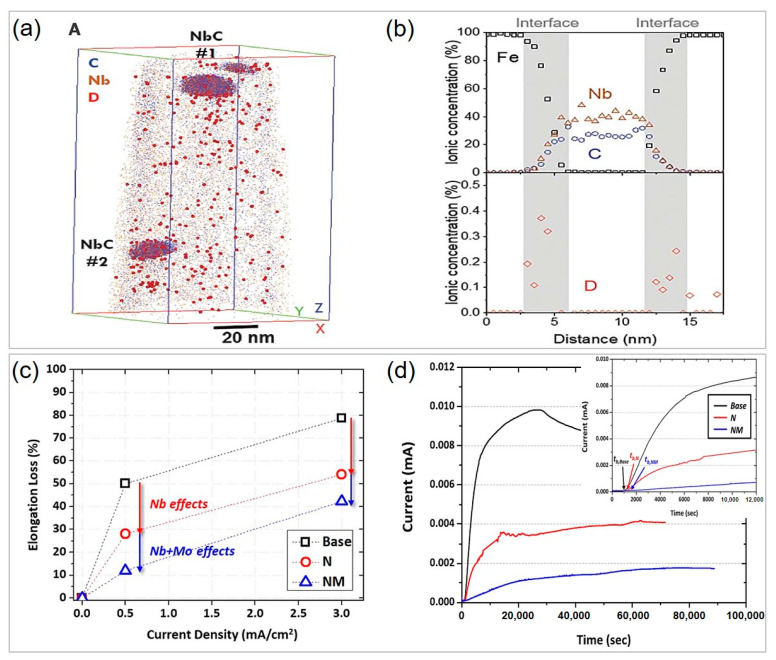
APT characterization of the nature of microalloying carbides as deep hydrogen trapping. (**a**) A 3D atom map showing the spatial distribution of D (large red spheres), C (small blue spheres), and Nb (small brown spheres) atoms, where D atoms are trapped by nanosized NbC particles. (**b**) Proximity histogram showing the composition variation across the precipitates NbC#1 in (**a**). Reprinted with permission from [71]. (**c**) Effects of Nb- or/and Mo- microalloying on the loss in elongation at hydrogen-charging current densities of 0.5 mA·cm^−2^ and 3 mA·cm^−2^ and on (**d**) hydrogen diffusivity of 32MnB5 steel. Reprinted with permission from [74].

In addition to the effect of segregation caused by microalloying elements, the formed carbides as hydrogen traps can also improve HE resistance. Hydrogen traps can be classified into reversible traps and irreversible traps in terms of binding energy [68]. Hydrogen atoms are unlikely to escape from irreversible traps due to high binding energy, while they are readily released into the matrix from reversible traps [69]. It is suggested that NaCl-structured carbides, such as NbC, TiC, and VC [70], are excellent irreversible hydrogen traps. Therefore, it is essential to design HE-resistant steels by fully understanding the interaction between hydrogen and microalloying carbides as irreversible hydrogen traps. Chen et al. [71] provided direct evidence that NbC/α-Fe incoherent interfaces can trap hydrogen through cryogenic APT technology. As shown in Figure 5a,b, the deuterium (D) atoms are located at the interfaces between NbC and α-Fe. There are a variety of defects in the interfaces, such as tetrahedral or octahedral interstitial sites, vacancies, and dislocations, all of which are possible hydrogen-trapping sites. Shi et al. [72] reported that the misfit dislocations in NbC/α-Fe semi-coherent interfaces are the hydrogen-trapping sites through high-resolution TEM, first-principles calculations, and thermal desorption spectroscopy analysis. Takahashi et al. [73] proposed that the hydrogen-trapping sites of VC are the carbon vacancies on the (001) crystal planes between the VC nanoprecipitate and the ferrite matrix. The irreversible hydrogen trapping caused by microalloying carbides can also reduce hydrogen diffusivity [70]. Jo et al. [74] found that multi-microalloying with Nb and Mo improved HE resistance in 32MnB5 steel more effectively than Nb addition alone (Figure 5c). The (Nb, Mo)C precipitates trapped hydrogen more effectively, resulting in the lowest hydrogen diffusivity, as observed in the hydrogen permeation curves in Figure 5d. Moreover, Mo as solute atoms can reduce the diffusivity of hydrogen atoms in steels [56], as the atomic size of Mo is larger than that of Fe, which produces a local strain field to attract hydrogen [75]. Therefore, microalloying carbides have been extensively applied to designing high-strength PHS. For instance, Zhang et al. [50] found that co-additions of Nb and Ti in 22MnB5 steel produced a large number of nanosized (Nb, Ti)C precipitates, increasing irreversible hydrogen-trapping sites and reducing HE susceptibility. Chen et al. [55] investigated that adding 0.14% V can form numerous precipitates in 30MnB5 steel, which significantly reduces hydrogen segregation at PAGB.

As discussed in Part 2.2, microalloying is a promising approach to refine the martensite structure, resulting in an increased proportion of grain boundary and a decreased hydrogen content per unit PAGB, which ultimately reduces HE susceptibility [76]. Yoo et al. [77] reported that 32MnB5 steel containing 0.03% Ti exhibits a finer prior austenite grain size (4.35 μm) compared to its 0.015% Ti-bearing counterpart (5.40 μm), and the former has better HE resistance. A similar observation was reported by Jo et al. [74].

Based on the aforementioned discussion, we present a summary of the effect of microalloying elements on the HE resistance of PHS in Table 3. Generally, higher hydrogen charging current density for a longer duration can result in more severe damage to ductility. For the separate addition of microalloying elements or Ta elements, the HE resistance is better with increased contents [52,78]. For example, when exposed to hydrogen charging at a solution of 3 wt% NaCl and 0.3 wt% NH_4_SCN for 5 h and 24 h, the elongation loss of 0.02% Ti-bearing 22MnB5 steel is 53.9% and 93.3%, which decreases to 15.1% and 38.4% for 0.03% Ti-adding counterpart, respectively [78]. For co-addition, increasing the content [50,77] and diversity [56,74] of microalloying elements can also achieve better HE resistance. For example, after hydrogen charging at 0.1 mol/L NaOH solution at a current density of 0.5 mA/cm^2^ for 24 h, the Nb- and Mo-bearing steel exhibits the smallest elongation loss of 12.2%, compared with Nb-bearing (28.8%) and reference steels (50.0%) [74]. However, adding microalloying elements may not always have a significant effect, as reported in Ref. [55].

### 2.4. Effect of Microalloying Elements on Other Service Performance of PHS

In addition to the commonly studied mechanical properties and HE, there are some investigations about the effect of microalloying elements on other service-related characteristics. These include bending properties, impact toughness, and hot ductility, among others. Examining the influence of microalloying elements on these properties can offer valuable insights for the design and optimization of materials with enhanced performance in real-world applications.

Most BIW parts bend and deform during collisions typically involving a state of close-to-plane-strain bending [37]. The bending property under these conditions is a critical parameter for evaluating the anti-collision ability of PHS parts, as well as their crash performance and fracture toughness of PHS. Tu et al. [79] reported that the co-addition of Nb and Mo can enhance both bending angles from 40.1° to 61.9° and bending energy from 44.2 J to 78.4 J. Yoo et al. [40] investigated that the 0.05% Nb addition, as well as 0.05% Nb and 0.1% Mo co-addition in 32MnB5 steel, can increase the bending angle from 56.4° to 60.0° and 69.8°, respectively. Järvinen et al. [39] found 0.15% Ti addition in 34MnB5 steel can improve the bending angle from 55° to 60.0°. In contrast, Pang et al. [80] found that Ti may deteriorate the bending properties by forming micron-sized TiN particles when the N content is not well controlled in 22MnB5 steel. Impact toughness is another parameter to identify the fracture toughness of PHS. Microalloying elements have also been shown to benefit impact toughness, as they refine the martensite structure and increase the cohesion of grain boundaries, thus suppressing crack propagation [40,48]. Additionally, microalloying carbides can strengthen the martensite matrix and reduce the risk of welding defects such as cold cracks, especially in PHS with high-carbon equivalents. In contrast, an increase in microalloying elements can have an adverse impact on the hot ductility of PHS as more precipitates restrict the plastic deformation at elevated temperatures, making the continuous casting and straightening processes more challenging [81].

Given the above discussions, we can briefly summarize the effect of microalloying elements on traditional Mn-B steel. For the traditional Mn-B steel, the effect of microalloying elements is highlighted: (1) Microalloying elements can refine the martensitic structure by both particles pinning and solute drag effect, leading to an appreciable precipitation strengthening effect. (2) Although the increase in microalloying elements cannot necessarily ensure the enhancement of strength–ductility synergy, particularly at higher total contents of microalloying elements, the addition of microalloying elements can significantly improve HE resistance. (3) The bending properties and impact toughness of PHS can also be improved by microalloying technology, but it may not be beneficial for hot ductility.

Despite these achievements, some actual problems remain unknown and are potential research areas in the future: (1) The effect of microalloying elements on mechanical properties or HE resistance needs quantitative analysis on an element-by-element basis. (2) To achieve an optimal combination of materials cost, service performance, and processing performance for PHS at different strength levels, the reasonable ranges of adding microalloying elements deserve further study.

## 3. Novel PHS Steels

### 3.1. Development Trend

The rapid development of PHS enables the diversification of material design. In contrast with traditional Mn-B steels, newly designed PHS incorporates additional alloying elements such as Si and Cr, and the thermal–mechanical processing is usually modified and optimized towards a multi-phase design. The incorporation of alloying elements enhances the hardenability of PHS, which can sometimes replace the use of B and can improve other service performances such as oxidation resistance. Furthermore, different processing methods can significantly affect the mechanical response of PHS. For example, quenching and partitioning (Q&P) and flash-partitioning processes have demonstrated their potential in tailoring the microstructure to achieve superior mechanical performance. Therefore, understanding the relationships between processing, microstructure, and properties is essential to comprehending the trends in the development of novel PHS.

### 3.2. Effect of Quenching and Partitioning Process on Mechanical Performance Improvement in PHS

**Figure 6:**
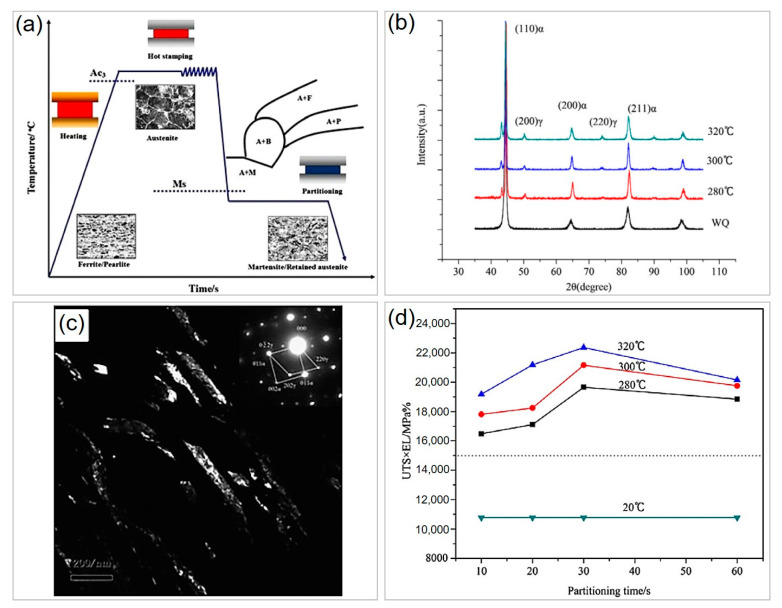
Enhancement of mechanical properties of PHS by introducing retained austenite via Q&P process. (**a**) Sketch map of Q&P process for press-hardened steels. (**b**) XRD patterns of the samples quenching at different partitioning temperatures. (**c**) Dark-field TEM image showing the morphology of retained austenite with an electron diffraction pattern (inset). (**d**) The variation in the product of strength and elongation corresponds to different quenching temperatures. Reprinted with permission from [83].

The traditional hot stamping process involves rapid quenching to room temperature to achieve a full martensite structure, as described in Figure 2a, but this approach typically results in reduced ductility despite high strength due to the single-martensite phase [82]. To solve the balance of strength–ductility synergy of PHS, Liu et al. [83] developed a Q&P process to link the phase transformation and C partitioning, which retains some austenite to improve ductility. Differing from directly quenching to room temperature after austenitizing (Figure 2a), the novel process first involves quenching the steel between the M_s_ and M_f_ points for a while to preserve a certain amount of austenite; in the meantime, C partitioning takes place at the insulation process, which improves the mechanical stability of retained austenite (Figure 6a). Finally, a mixture of martensite + retained austenite can be obtained when further cooling to ambient temperature (Figure 6b), and the nanosized retained austenite has a film-like morphology (Figure 6c). The volume fraction and mechanical stability of retained austenite can be tailored by changing the partitioning temperatures (280–320 °C) and durations (10–60 s), leading to a significant improvement in the combination of strength and ductility compared to the single-martensite phase by directly quenching to 20 °C (Figure 6d). It should be noted that the Si content of the novel PHS (0.22C-1.58Mn-0.81Si-0.022Ti-0.0024B) is much higher than that of 22MnB5 steel (normally 0.2–0.4% shown in Table 1), which suppresses the formation of cementite and triggers C partitioning to untransformed austenite. More recently, Linke et al. [84] systematically studied the effect of Si content (0.25%, 0.5%, 0.78%, and 1.5%) on retained austenite and mechanical performance of 22MnB5 steels subjected to both one-step and two-step Q&P heat treatment processes. The results showed that higher Si content can increase both the volume fraction and the content of retained austenite, which contributes to enhanced mechanical properties. Zhu et al. [85] systematically investigated the influence of quenching temperature 250–324 °C) on a novel PHS with 1.6% Si, and found that quenching to 290 °C can achieve the maximum amount of retained austenite, which results in the best strength–ductility balance. Seo et al. [86] found that both additions of 1.6% Si and 1% Cr in PHS can achieve a higher content of austenite than the 1.6% Si-containing counterpart as Cr can stabilize austenite, which leads to the enhancement in strength–ductility trade-off. Liang et al. [87] found that increasing partitioning duration at 260 °C can enhance the mechanical performance of a 1.5% Si- and 0.9% Cr-containing 38MnB5 steel, and the maximum strength–ductility synergy of 23 GPa% is achieved at prolonged partitioning time of 600 s due to the enhanced TRIP effect.

In recent years, there has been an increasing number of studies on the Q&P process of PHS, such as intercritical deformation + Q&P process [88,89], hot air partitioning [90], non-isothermal partitioning [91], and multi-scale coupling simulation [92]. However, it should be noted that the Q&P process is difficult to apply in the hot-stamping production line. The steel sheet needs to be transferred to a heated die with fixed temperatures after austenitizing, and then be formed and partitioned, which is challenging to control accurately and inevitably reduces production efficiency. To introduce the retained austenite effectively under industrial conditions, a more advanced technology known as the flash-partitioning process has been proposed.

### 3.3. Effect of Flash-Partitioning Process on Mechanical Performance Improvement in PHS

**Figure 7:**
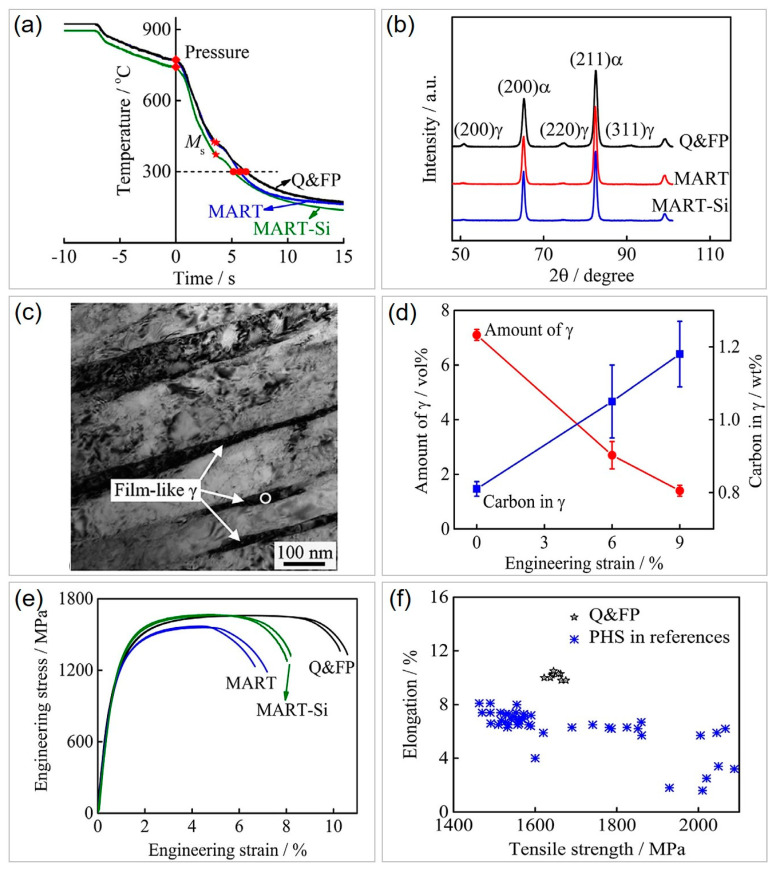
Enhancement of mechanical properties of PHS by introducing retained austenite via the flash−partitioning process. (**a**) Variation in temperature with time during the flash−partitioning process for Q&FP, MART, and MART−Si steels. (**b**) XRD patterns of experimental steels after the flash-partitioning process. (**c**) Bright-field TEM image showing film-like retained austenite in Q&FP steel. (**d**) Progressive austenite-to-martensite transformation during tensile tests in Q&FP steel. (**e**) Mechanical response of experimental steels. (**f**) Enhanced ductility induced by retained austenite of Q&FP steel. Reprinted with permission from [14].

Similar to the materials in Q&P heat treatment, the flash-partitioning process also adopts a high-Si strategy to stabilize the austenite [14]. The cooling rate drops when the press-hardening process is carried out on a flat die instead of a hot-stamping die. As depicted in Figure 7a, the cooling rate at the commencement of pressing is around 100 °C/s, and significantly drops when martensitic transformation (M_s_ points) occurs due to the release of latent heat. This decline in cooling rate affords sufficient time for dynamic carbon diffusion from transformed martensite to untransformed austenite. It is worth noting that Si-alloyed Q&FP (Fe-0.25C-0.24Mn-1.53Si) steel has higher M_s_ temperatures than MART-Si (Fe-0.25C-2.68Mn-1.74Si) and MART steels (Fe-0.25C-0.21Mn). This characteristic imparts higher austenite stability and more time for C partitioning. As a consequence, retained austenite with a volume fraction of 7% is observed in Q&FP steel, in contrast to very limited austenite in MART-Si and MART steels (Figure 7b). The film-like retained austenite with a nanometer size in Q&FP steel is incorporated within martensite lath (Figure 7c). Despite most metastable austenite transforming progressively into martensite during tension [93,94], the remaining austenite with a high C content is relatively stable (Figure 7d). The strain-induced martensitic transformation can significantly enhance the work hardening and ductility of the Q&FP steel, as compared to the MART-Si and MART steels with the same or lower stress levels (Figure 7e). Therefore, the flash-partitioning process represents a significant breakthrough in toughening PHS, as it can improve the ductility of PHS to above 10% (Figure 7f). It is noteworthy that distinct from the Q&P process, the flash-partitioning process shows potential for application in the hot-stamping production line.

### 3.4. Effect of Si and Cr Alloying on Mechanical Performance and Oxidation Resistance Improvement in PHS

**Figure 8:**
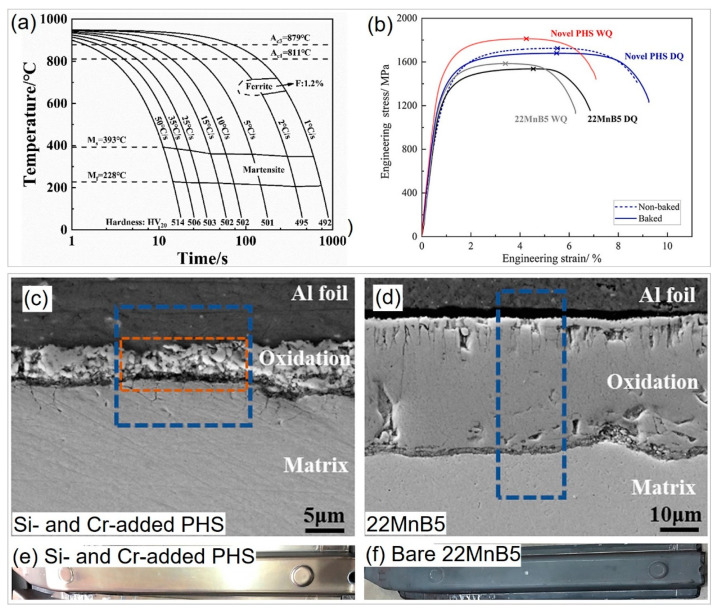
Effect of Si and Cr alloying on the hardenability, mechanical properties, and oxidation resistance of press-hardened steels. (**a**) Continuous cooling transition (CCT) diagram of Cr- and Si-alloyed press-hardened steels. Reprinted with permission from [37]. (**b**) Mechanical properties of Cr- and Si-alloyed press-hardened steels, compared with traditional 22MnB5 steel. Reprinted with permission from [13]. (**c**,**d**) SEM secondary electron images of the oxide layer of (**c**) Cr- and Si-added PHS and (**d**) 22MnB5 steel after soaking at 930 °C for 120 s in air. Reprinted with permission from [95]. (**e**,**f**) Surface qualities of (**e**) Cr- and Si-added PHS and (**f**) bare 22MnB5 press-hardened steel after hot stamping. Reprinted with permission from [98].

In recent years, researchers have focused on optimizing the oxidation-resistant ability of press-hardened steel (PHS) in addition to improving its mechanical performance by adding more Si and/or Cr and tuning the thermomechanical processing routes. Xu et al. [13,37,95] developed a novel PHS with high Si and Cr content, satisfying Si + Cr ≤ 4% to eliminate the use of B. As shown in the continuous cooling transformation diagram in Figure 8a, the Cr and Si content enables a critical cooling rate of 5 °C/s for complete martensitic transformation, which is much lower than the critical cooling rate of 27 °C/s for 22MnB5 steel [6,37]. Mohrbacher et al. [96] reported that the increase in C, Mn, Cr, and Mo can trigger martensitic transformation by postponing ferrite and pearlite formation in 22MnB5 steel. Lee et al. [97] found that increasing the Cr content to 1.2% and above in B-free 22MnB5 steel can achieve a fully martensitic microstructure at a cooling rate of 50 °C/s, as Cr is capable of dragging the phase boundary of austenite and martensite during phase transformation. Thus, a high Cr value is verified to enhance the hardenability and replace B.

Moreover, the novel PHS with high Si and Cr content also exhibits superior mechanical performance compared to traditional 22MnB5 steel despite quenching conditions (Figure 8b). Wei et al. [13] attributed this to the solid-solution strengthening of Si, which enhances strength, and the increase in Si and Cr that contributes to the C partitioning from martensite to austenite and preserves 4.3 vol.% retained austenite during die quenching, which is responsible for the improved ductility. Meanwhile, Hou et al. [95] found that the Si- and Cr-alloyed PHS also become more oxidation resistant (Figure 8c,d). The thickness of the oxide layer of Si- and Cr-containing PHS (5 μm) is much thinner than that of 22MnB5 steel (36 μm) after soaking at 930 °C for 120 s in air. The formed Si- and Cr-rich oxide layer further suppresses oxygen diffusion and reaction with the inner matrix, resulting in good oxidation resistance. As a result, the surface of Si- and Cr-added PHS is cleaner and smoother than that of 22MnB5 steel (Figure 8e,f), enabling it to be coating free [98]. Bare 22MnB5 steel is vulnerable to severe oxidation during the transfer from the heating furnace to the die and the hot stamping process in the die, which seriously affects surface quality, weldability, and paintability. Additional shot or sandblasting is needed to remove the oxide scale [9,18]. On the other hand, Al-Si- or Zn-based coatings are alternative strategies to protect the surface from oxidation and decarbonization, but this can also significantly increase the production costs of PHS, including coating costs and a premium for the intellectual property of coating [9]. Additionally, the coating may hurt weldability [99,100]. Although the increase in Si and Cr contents increases the material cost to a certain extent, it is much lower than the coating cost. Thus, the Si- and Cr-alloyed PHS is a significant breakthrough to obtaining coating-free PHS products.

### 3.5. Effect of Intercritical Temperature Quenching on Mechanical Performance and Oxidation Resistance Improvement in PHS

**Figure 9:**
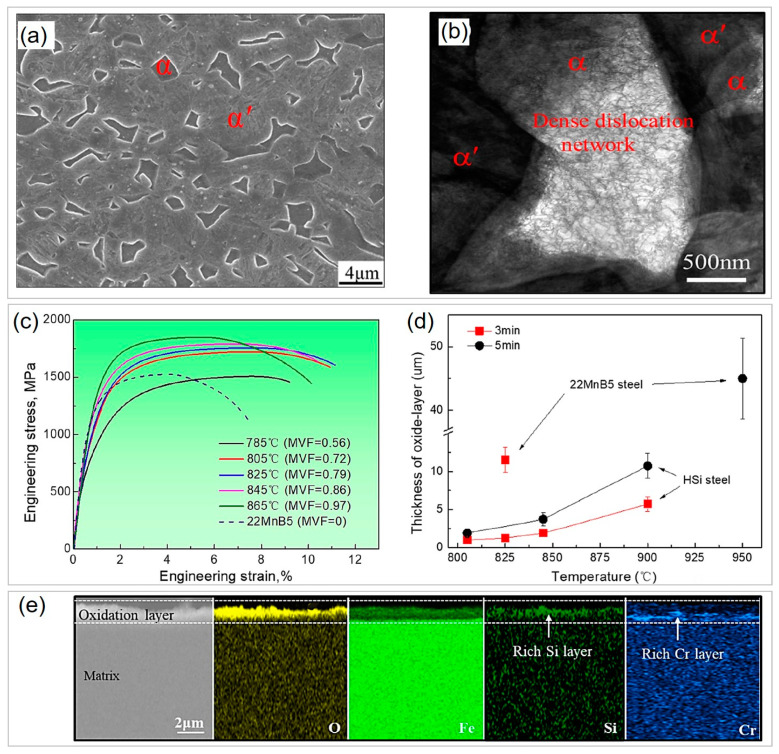
Enhancement of mechanical properties and oxidation resistance via dual-phase (hard martensite + soft ferrite) design in press-hardened steels produced by intercritical temperature quenching. (**a**) Typical SEM secondary electron image of martensite (α’) and ferrite (α) dual-phase microstructure and (**b**) TEM image showing the dislocation structure when quenching from 825 °C. (**c**) Tensile curves of studied press-hardened steels subjected to quenching at different intercritical temperatures compared with 22MnB5 steel. (**d**) Comparisons of oxidation resistance of dual-phase press-hardened steels with 22MnB5 steel at different temperatures and durations. (**e**) SEM secondary electron and corresponding energy dispersive X-ray spectroscopy images showing the formation of dense Si- and Cr-rich oxide layer for dual-phase press-hardened steels. Reprinted with permission from [101].

The production of partial martensitic structures by quenching from the austenite + ferrite zone is another practice in the manufacturing of PHS. For example, Ding et al. [101] proposed a newly developed PHS (Fe-0.25C-1.29Mn-1.58Si-0.56Cr-0.04Nb) with martensite + ferrite structure when quenching at intercritical temperature covering a wide range, from 865 °C to 766 °C. When decreasing temperature from 865 °C to 785 °C, the volume fraction of ferrite increases from 2.9% to 42.2%. The typical martensite + ferrite microstructure after quenching from 825 °C is shown in Figure 9a. The relatively clean ferrite is embedded inside the hierarchical martensite matrix with a volume fraction of 79.5%, which is different from traditional dual-phase steel where ferrite accounts for most of the volume fraction [102,103]. The TEM image shows that ferrite has a dense dislocation network, and the dislocation density seems to be higher at the interphase to accommodate the strain gradient between ferrite and martensite (Figure 9b). The tensile curves indicate that the increase in ferrite can improve the ductility without significant sacrifice of strength when quenching from 805 °C to 845 °C, while the ductility is compromised upon, further decreasing the quenching temperature to 785 °C due to the formation of brittle martensite with a high C content of 0.44% (Figure 9c). The highest product of tensile strength and total elongation, 19.7 GPa, is obtained upon quenching at 825 °C, which is superior to that of 22MnB5 steel. The straining capability of ferrite is responsible for increased ductility. In contrast, quenching at intercritical temperature fails to significantly enhance the mechanical properties of 22MnB5 steel [104], indicating the importance of optimizing the composition. In addition to the superior mechanical performance, the designed dual-phase PHS is also more oxidation resistant than 22MnB5 steel (Figure 9d). Due to the lower austenitizing temperature and shorter soaking time for quenching at intercritical temperature, the oxide layer thickness of present dual-phase PHS is much thinner than 22MnB5 steel. Additionally, a dense Si- and Cr-rich oxide layer is observed due to the relatively high Si (1.58%) and Cr (0.56%) content of PHS, which can impede further reaction between the matrix and oxygen similar to Ref [95]. Yi et al. [105] also investigated a novel dual-phase PHS with a ferrite + martensite structure, which exhibits an extremely high work-hardening ability and achieves a strength of 1550 MPa and total elongation of 9%.

### 3.6. Effect of Other Processes on the Mechanical Performance of PHS Compared with Commercial Automotive Steels

**Figure 10:**
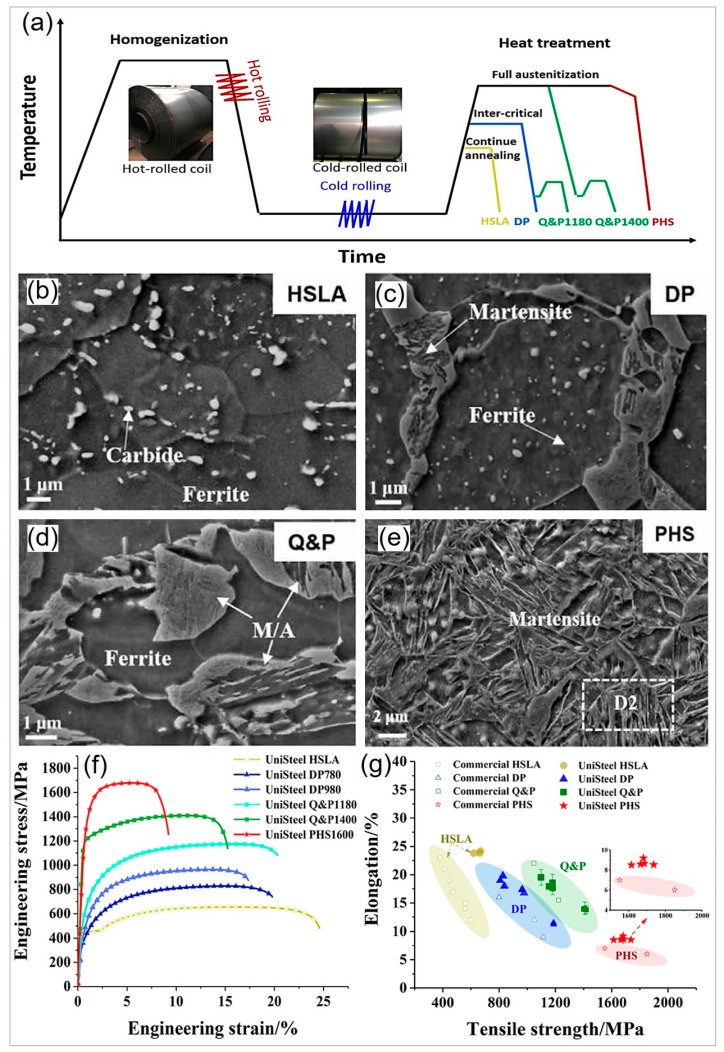
Multi-phase tailoring of Cr- and Si-alloyed PHS. (**a**) Application of various thermal mechanical processing to Cr- and Si-alloyed press-hardened steel, to fabricate variant grades such as high-strength low alloy (HSLA), dual-phase (DP), quench and partitioning (Q&P), and press-hardened steels (PHS). SEM secondary electron images showing microstructures of (**b**) HSLA steel, (**c**) DP steel, (**d**) QP steel, and (**e**) PHS. (**f**) Mechanical properties of the variant grades. (**g**) Comparison of the tensile strength and elongation for variant grades and corresponding commercial steel grades. Reprinted with permission from [106].

Based on the above discussion, it can be concluded that the multi-phase design can improve the mechanical properties of PHS. Recent advancements in the Uni-Steel concept have led to a significant expansion in the microstructures and properties of PHS [106]. The chemical composition of the steel used in the study (Fe- < 0.25C-1.1Mn- < 2.5Cr- < 0.25Si-0.025Nb) is also comparable to Si- and Cr-bearing novel PHS [13]. After undergoing hot and cold rolling, the steel was subjected to various thermomechanical processing routes, resulting in microstructures similar to those found in traditional HSLA, DP, Q&P, and PHS steels (Figure 10a–e). For example, subcritical annealing at 780 °C for 8 min during continuous annealing processing (Figure 10b) was used to obtain the HSLA microstructure, which consists of dispersed carbides embedded in a ferrite matrix. Conversely, die quenching after austenitizing at 920 °C for 6 min and tempering at 170 °C for 20 min (Figure 10e) was used to achieve the PHS structure, which has a fully martensitic structure with some carbides. As a result, a wide range of strength and ductility can be achieved, allowing for the production of BIW parts tailored for meeting specific requirements. Additionally, the variant grades of the newly designed steel exhibit improved mechanical performance than commercial steel grades (Figure 10f,g). The study demonstrates that the Si- and Cr-alloyed novel PHS can be utilized to fabricate various BIW parts by customized thermomechanical processing, thereby confirming the efficacy of the multi-phase design of PHS illustrated in Figure 1e.

For the novel PHS, the relationship between material design, processing, multi-phase microstructures, and mechanical response is fully understood. The novel PHS shows potential in industrial applications due to appealing mechanical properties and other service performance such as oxidation resistance.

Despite these achievements, some potential research fields are highlighted: (1) Research focuses on the effect of high Si and Cr contents on improving oxidation resistance, but whether the obtained thin oxide layer can satisfy the subsequent painting processing to fabricate coating-free PHS products is unknown. (2) Adapting the novel processing technology to the hot-stamping production line deserves more attention, including quenching and partitioning (Q&P), flash-partitioning process, and intercritical temperature quenching. (3) More research on other service performances of newly developed PHS such as formability [107,108], weldability [109], fatigue [110], wear [111], corrosion [112,113], HE resistance [114], and bending properties [91] is recommended to promote their use in the future. (4) Except for traditional C-Mn steels or novel PHS, other steel grades, such as medium-Mn steels [115,116,117,118] and stainless steels [119], are also suitable for hot stamping, and these innovative topics have the potential to expand the application prospects of hot stamping and in-depth research.

## 4. Comparison of the Mechanical Properties of Novel PHS and Traditional Mn-B Steel

Table 4 summarizes the mechanical properties of some of the novel PHS compared with traditional 22MnB5 steel. For the materials design of novel PHS, more alloying elements such as Si [13,14,83,91,101], Cr [13,91,101], Ni [101], and Al [105] are introduced. The composition design has unique advantages in improving mechanical properties. For example, novel Si- and Cr-added PHS has 200 MPa higher strength than 22MnB5 steel with similar C content, under the same processing conditions of water quenching or flat die quenching, accompanied by better ductility [13]. The specific reasons for the enhanced mechanical performance of PHS by tailoring chemical compositions deserve further study. Additionally, toughening PHS by introducing retained austenite or ferrite by innovative processing technology such as the Q&P process [83,91], flash-partitioning process [13,14], and intercritical temperature quenching [101,105] is effective. For instance, the total elongation increases from 6.6% to 10.3% at a limited sacrifice of the strength of 31 MPa, when the Q&P process is applied to introduce a 5% volume fraction of retained austenite in the martensite matrix [83]. The flash-partitioning process technology also promotes the ductility of 1500 MPa grade PHS to a remarkable level of 10.4% [14], much higher than the general elongation of 7% of traditional 22MnB5 steel [91]. The novel PHS fabricated by intercritical temperature quenching can achieve a high strength of 1843 MPa and an elongation of 9.8%, much higher than 22MnB5 steel (1522 MPa and 7.4%) [101].

## 5. Conclusions and Future Outlook

This review principally introduces relevant studies on the multi-scale microstructure tailoring of traditional Mn-B steels and novel PHS. The main focus is on the effect of chemical composition and thermomechanical processing on related mechanisms for mechanical properties and other service performance. The optimization of chemical composition and processing provides various options for PHS materials tailored to specific application scenarios. This potentially enables multi-scale microstructure tailoring including precipitation, segregation, grain boundary, multi-phase design, etc., which significantly enhances the mechanical properties. The review also presents the overall development trend in PHS.

(1) One critical development trend in PHS is a superior combination of strength and ductility to satisfy the increasing demands for lightweight BIW. With this purpose, composition and process can be reasonably optimized, subject to cost constraints. As aforementioned in the review, PHS materials with high strength, excellent ductility, and toughness have proliferated in recent years.

(2) On the other hand, considering that carbon emission and energy consumption during steel production are critical components of the entire vehicle lifecycle, near-net production processes, such as compact strip production, are being explored to produce PHS ecologically at a reduced cost during the raw material acquisition stage.

(3) In addition to traditional hot stamping, low-temperature hot stamping technologies for novel steels are in demand which can significantly reduce energy consumption. By adopting these approaches, the automotive industry can achieve sustainable development in the future.

## Figures and Tables

**Table 2:** Effect of microalloying elements on microstructure and mechanical properties of PHS.

Steel Type	Increased Additions of Microalloying Elements	Microalloying Elements					Prior Austenite Grain Size (μm)	Ultimate Tensile Strength Change (MPa)	Total Elongation Change (%)	Refs.
		Ti	Nb	V	Mo	Ta				
22MnB5	Reference	/	/	/	0.145	/	18.45	1673	13.09	[49]
	+Nb	/	0.027	/	0.144	/	10.01	+59	+0.73	
	+Nb	/	0.049	/	0.140	/	6.88	+57	+0.91	
22MnB5	Reference	0.03	/	/	/	/	16.98	1681.8	7.9	[50]
	+Nb	0.031	0.023	/	/	/	9.03	+15.1	+0.4	
	+Nb	0.029	0.072	/	/	/	7.12	+57	+0.6	
22MnB5	Reference	0.03	/	/	/	/	16.4	1663	/	[54]
	+Nb	0.031	0.022	/	/	/	9.8	+38.2	/	
	+Nb	0.033	0.053	/	/	/	6.7	+3	/	
	+Nb	0.029	0.078	/	/	/	7.0	−88.5	/	
22MnB5	Reference	0.04	/	/	/	/	11.7	1538.7	6.0	[51]
	+Nb+V	0.04	0.04	0.042	/	/	7.6	+32.1	+0.6	
22MnB5	Reference	/	/	/	/	/	17.16	1708	7.2	[52]
	+Ta	/	/	/	/	0.026	9.55	+21	+0.1	
	+Ta	/	/	/	/	0.053	6.73	+30	+0.2	
30MnB5	Reference	0.036	/	/	/		8	1817	8.3	[34]
	+Nb	0.07	0.046	/	/		6.3	+369	−2.7	
	+Nb+V	0.063	0.048	0.14	/		3.3	0	−2	
	+Nb+V	0.034	0.021	0.65	/		1.9	−215	−0.5	
30MnB5	Reference	0.07	0.046	/	/		6.5	2100	9.7	[55]
	+V	0.068	0.068	0.14	/		3.6	−247	−1.1	
32MnB5	Reference	0.03	/	/	/		8.98	2070	8.7	[40,56]
	+Nb	0.03	0.05	/	/		4.65	−166	−0.6	
	+Nb+Mo	0.03	0.05	/	0.1		4.22	−146	−1	
	+Nb+Mo	0.03	/	/	0.15		8.27	+50	+1.4	
38MnB5	Reference	/	/	/	0.16		17	2011	5.92	[53]
	+Nb	/	0.054	/	0.16		11	+168	+0.72	

**Table 3:** Effect of microalloying elements on hydrogen embrittlement resistance of PHS.

Steel Type	Increased Additions of Microalloying Elements	Microalloying Elements					Hydrogen Charging Conditions		Strain Rate	Elongation Loss (%)	Ref.
		Ti	Nb	V	Mo	Ta					
22MnB5	Reference	0.030	/	/	/	/	0.5 mol/L H_2_SO_4_ and 0.25 g/L CH_4_N_2_S	1 mA/cm^2^, 1 h	6.6 × 10^−5^ s^−1^	57.1	[50]
	+Nb	0.031	0.023	/	/	/				52.0	
	+Nb	0.029	0.072	/	/	/				44.7	
22MnB5	Reference	0.02	/	/	/	/	3 wt% NaCl and 0.3 wt% NH_4_SCN	5 mA/cm^2^, 5 h	2.4 × 10^−5^ s^−1^	53.9	[78]
								5 mA/cm^2^, 24 h		93.3	
	+Ti	0.03	/	/	/	/		5 mA/cm^2^, 5 h		15.1	
								5 mA/cm^2^, 24 h		38.4	
22MnB5	Reference	/	/	/	/	/	0.5 mol/L H_2_SO_4_ and 0.25 g/L CH_4_N_2_S	0.5 mA/cm^2^	9.5 × 10^−5^ s^−1^	69.4	[52]
	+Ta	/	/	/	/	0.026				42.5	
	+Ta	/	/	/	/	0.053				33.8	
30MnB5	Reference	0.07	0.046	/	/	/	0.1 mol/L NaOH	5 mA/cm^2^, 24 h	1 × 10^−5^ s^−1^	87.1	[55]
	+V	0.068	0.048	0.14	/	/				82.1	
32MnB5	Reference	0.03	/	/	/	/	0.1 mol/L NaOH	0.5 mA/cm^2^, 24 h	1 × 10^−4^ s^−1^	50.0	[56]
								3 mA/cm^2^, 24h		78.8	
	+Mo	0.03	/	/	0.15	/		0.5 mA/cm^2^, 24 h		15.9	
								3 mA/cm^2^, 24 h		26.6	
32MnB5	Reference	0.03	/	/	/	/	0.1 mol/L NaOH	0.5 mA/cm^2^, 24 h	1 × 10^−4^ s^−1^	50.0	[74]
								3 mA/cm^2^, 24 h		78.8	
	+Nb	0.03	0.05	/	/	/		0.5 mA/cm^2^, 24 h		28.8	
								3 mA/cm^2^, 24 h		53.8	
	+Nb + Mo	0.03	0.05	/	0.10	/		0.5 mA/cm^2^, 24 h		12.2	
								3 mA/cm^2^, 24 h		42.2	
32MnB5	Reference	0.015	0.05	/	0.1	/	0.1 mol/L NaOH	0.5 mA/cm^2^, 24 h	1 × 10^−4^ s^−1^	62.1	[77]
								3 mA/cm^2^, 24 h		78.2	
	+Ti	0.03	0.05	/	0.1	/		0.5 mA/cm^2^, 24 h		12.2	
								3 mA/cm^2^, 24 h		42.2	

**Table 4:** Comparison of mechanical properties of novel PHS and traditional Mn-B steel.

Steel Type	Chemical Composition	Processing	Gauge Length (mm)	Ultimate Tensile Strength (MPa)	Total Elongation (%)	Ref.
Novel PHS	0.22C-1.58Mn-0.81Si- 0.022Ti-0.0024B	Quenching to room temperature	10	1632	6.6	[83]
		Q&P process		1601	10.3	
				1576	11.3	
				1522	12.6	
				1569	13.5	
				1510	14.8	
				1562	12.9	
Novel PHS	0.23C-1.1Mn-(Cr + Si) <5	Hot stamping	50	1668	7.1	[91]
		Hot stamping + Q&P process		1590	7.6	
22MnB5	Al-Si coated 22MnB5	Flat die quenching		1548	6.8	
Novel PHS	0.25C-0.24Mn-1.53Si	Flat die quenching (flash-partitioning process)	50	1661	10.4	[14]
Novel PHS	<0.25C-1.1Mn- < 2.5Cr- < 2.5Si-0.025Nb	Water quenching	50	1794	6.5	[13]
		Flat die quenching (flash-partitioning process)		1735	8.5	
22MnB5	0.23C-1.2Mn-0.16Cr-0.22Si-0.005B	Water quenching		1592	6.1	
		Flat die quenching		1534	6.8	
Novel PHS	0.25C-1.29Mn-1.58Si-0.56Cr-0.27Ni-0.038Nb	Intercritical temperature quenching	25	1720	10.8	[101]
				1762	11.2	
				1796	10.3	
				1835	9.8	
				1843	9.8	
22MnB5	0.22C-1.35Mn-0.28Si-0.21Cr-0.03Ti-0.04Mo	Oil quenching from 950 °C		1522	7.4	
Novel PHS	0.4C-2.02Mn-0.26Si-2.5Al	Intercritical temperature quenching	10	1539	8.8	[105]
